# Balancing Strength and Cell Viability in Gelatin Methacrylate/Gellan Gum Bioink Formulations

**DOI:** 10.1021/acsomega.5c09665

**Published:** 2026-03-03

**Authors:** Eduardo H Backes, Leonardo A Pinto, João F Gomes Neto, Tainara P Lima Lima, Pedro L Granja, Luiz A Pessan, Marimélia A Porcionatto

**Affiliations:** † Federal University of São Carlos (UFSCar), Graduate Program in Materials Science and Engineering (PPGCEM), São Carlos 13565-905, Brazil; ‡ Federal University of São Carlos (UFSCar), Department of Materials Engineering, São Carlos 13565-905, Brazil; § Instituto de Investigação E Inovação Em Saúde (i3S), i3S - Universidade Do Porto/Biofabrication Group, Porto 4200-135, Portugal; ∥ Universidade Federal de São Paulo (UNIFESP), Laboratory of Molecular Neurobiology, Escola Paulista de Medicina, São Paulo 04023-062, Brazil; ⊥ Universidade Federal de São Paulo (UNIFESP), Department of Biochemistry, Escola Paulista de Medicina, UNIFESP, São Paulo 04023-062, Brazil

## Abstract

Soft tissue injuries resulting from trauma or degeneration are challenging to treat due to limited regenerative capacity, particularly in complex tissues, such as the central nervous system (CNS), nerves, and cartilage, where biomechanical and biochemical factors hinder effective repair. In these cases, tissue engineering presents a promising approach by combining biomaterials, cells, and bioactive signals to enhance soft tissue regeneration; however, its success relies on the compatibility between implanted materials and native tissue. Among the advances in this field, 3D bioprinting enables precise spatial control of the scaffold architecture and cell positioning, making it well-suited for developing constructs that mimic native tissue. In this study, we developed and characterized a series of bioink formulations based on a dual network system of gelatin methacrylate (GelMA) combined with gellan gum (GG). The GelMA/GG hydrogels were evaluated using rheological and compression testing as well as biodegradation and cell viability assays, including live/dead fluorescence microscopy. Formulations containing two different concentrations of GelMA (2.5 and 4.0% w/w) and GG (0.25 and 0.50% w/w) were tested, and the rheological results showed a strong dependence of the elastic component (*G*′) on GG concentration. For the 2.5% GelMA formulations, increasing the GG content significantly enhanced the Young’s modulus. In 4.0% GelMA formulations, stiffness increased as the GG concentration rose. Higher GG content decreased biodegradation over 14 days in phosphate-buffered saline and reduced cell viability due to the hydrogel’s increased stiffness. The bioinks demonstrated suitable rheological properties for bioprinting, achieving over 98% cell viability after 1 day. Additionally, formulations such as 4.0% GelMA with 0.25% GG and 2.5% GelMA with 0.5% GG exhibited high cell viability (above 85%) when maintained even after longer culture periods, such as 14 days. These results indicate that GelMA/GG hydrogels have great potential as versatile, tunable bioinks for soft-tissue engineering in the CNS. Future research will focus on modifying the hydrogel network’s rigidity to enhance cell viability further and refine its application in bioprinting strategies for regenerating soft tissues in the central nervous system.

## Graphical abstract

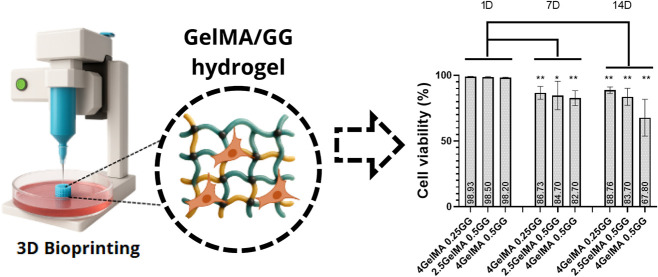

## 1 Introduction

Traumatic and degenerative injuries affect various human soft tissues, including neural, muscular, vascular, and cartilaginous tissues, and significantly increase the global health burden. These injuries involve social and economic expenses and, in severe cases, contribute to long-term disabilities and even mortality.^1,2^ Despite their self-repairing and regenerative capabilities, tissues often fail to adequately restore the original functional architecture.^3^ The natural regenerative limitation becomes even more critical in complex tissues with high structural and functional specialization, such as the central nervous system (CNS), nerves, and cartilage, where biomechanical and biochemical complexity impose additional barriers to tissue repair.^4^

In such cases, advanced therapeutic strategies, including stem cell transplantation and tissue engineering, have emerged as promising alternatives to overcome the limitations of natural repair. In the CNS, the restricted capacity for neurogenesis in adulthood hampers neuronal replacement after injury.^5^ Similarly, in cartilage, high hydration, low stiffness, and a dependence on specific biochemical cues also compromise self-regeneration. Beyond the direct clinical use, the reconstruction of soft tissues has gained increasing relevance in the development of biomimetic *in vitro* models for preclinical research and pharmaceutical testing.^6^ In addition, the current demand for not only physiologically but also ethically representative alternatives to animal models underscores the importance of creating platforms that accurately replicate the mechanical and biochemical characteristics of native tissues, thereby enabling safer and more cost-effective platforms for testing drugs and therapies.

The effectiveness of these regenerative approaches largely depends on the adequate recreation of the tissue microenvironment in which the cells will be placed. Mechanical properties, including stiffness, viscoelasticity, and structural integrity, interact synergistically with biochemical signals to modulate cellular functions such as adhesion, proliferation, migration, and differentiation.^7^ However, injured areas often present a hostile environment, both mechanically and biochemically, impairing cell survival and new tissue formation.^8^ Therefore, developing biomaterials that can mimic the properties of native tissue is vital not only to encourage functional recovery but also to facilitate mechanistic studies in physiologically relevant conditions.^9^

3D bioprinting is a leading technology for developing biomaterials, effectively enabling the creation of biomimetic structures with precise control over geometry and cell positioning.^10–12^ However, this technique still faces limitations due to strict requirements for bioinks, which must simultaneously provide a soft, suitable microenvironment for cells while maintaining structural fidelity after printing and during maturation, when necessary.^13–15^ This scenario highlights a design challenge: hydrogels that support high cell viability and cellular function often lack the mechanical strength required for high-resolution printing and structural stability. Additionally, problems such as decreased cell viability after printing and inadequate cell-material interactions persist, hindering their clinical use.

In this context, gelatin methacrylate (GelMA) emerges as a promising alternative to address some of these limitations.^16^ GelMA is a photo-cross-linkable hydrogel derived from gelatin, whose composition offers high biochemical similarity to the native extracellular matrix due to the presence of RGD (Arg-Gly-Asp) peptide sequences.^17^ Another particularly relevant aspect is that the mechanical behavior of GelMA-based hydrogels can be precisely modulated not only to reproduce the profile of the target tissue but also to enable self-regeneration during the printing process.^18^ However, at low concentrations, GelMA exhibits low viscosity and rapid degradation, compromising its printability and structural stability, and limiting its application in extrusion-based 3D bioprinting.^19^ To address these limitations, strategies beyond simply increasing polymer concentration or cross-linking density have been proposed, such as adding viscosity enhancers, incorporating reinforcing agents, or developing bioinks as a dual-network system combining GelMA with other hydrogels.^20–22^

Among the materials that can be combined with GelMA, gellan gum (GG) stands out as an anionic, water-soluble polysaccharide produced by the fermentation of *Sphingomonas paucimobilis*.^23^ Recently approved by the US Food and Drug Administration (FDA), GG has been proposed for several applications, particularly in cartilage regeneration, due to its high biocompatibility, biodegradability, good mechanical properties, and low cytotoxicity.^24,25^ However, when used alone, GG presents challenges, including a relatively high gelation temperature and low cell adhesion sites, which justify its use in combination with other hydrogels or composite formulations to enhance its properties.^26,27^

Previous studies have already highlighted the potential of combining GelMA and GG for soft tissue engineering. For example, Zhuang et al.^28^ developed a UV-assisted, layer-by-layer bioprinting strategy for fabricating complex 3D structures from GelMA/GG bioinks. Minimal GG addition (0.1–0.2% w/v) improved printability without compromising biocompatibility. Optimal viscosity ranges were established for printing and cell encapsulation, and the effect of UV exposure on the resolution and cell viability was assessed. This approach yielded constructs with excellent geometric fidelity and mechanical stability, and the strategy can be applied to other photocurable materials. Mouser et al.^29^ also investigated GelMA/GG hydrogels as bioinks for cartilage bioprinting, testing concentrations of 3–25% w/v GelMA and 0–1.5% w/v GG. They evaluated printability, rheological properties, stiffness of photocured constructs, and the chondrogenic potential of encapsulated chondrocytes. GG incorporation improved filament deposition by inducing yielding behavior, increasing construct stiffness, and promoting chondrogenesis. However, higher GG concentrations impaired cartilage matrix production and its homogeneous distribution, and even higher levels hindered cell encapsulation due to excessive yield stress.

GG/GelMA bioinks combine strong ionotropic gelation with favorable shear-thinning and high shape fidelity under physiological conditions, enabling dual (ionic + photo-cross-linking) control of stiffness and printing performance.^8,30^ Compared with alginate, xanthan, guar, and carrageenan, GG generally offers a better balance between viscosity and printability, a more robust structure at low concentrations, and broad biocompatibility in pharmaceutical and tissue engineering formulations.^31^ Despite recent advances, systematic studies assessing the influence of GelMA/GG composition on rheological and mechanical properties, geometric fidelity, and cell response in soft tissue bioprinting remain scarce. Therefore, this work focuses on developing and characterizing GelMA/GG bioink formulations with properties tailored for 3D bioprinting. The effects of composition are analyzed to ensure printing precision, adequate rheological and mechanical properties, and biocompatibility, contributing to the development of 3D bioprinted scaffolds for advanced soft tissue engineering.

## 2 Materials and Methods

### 2.1 Hydrogel Preparation

GelMA was synthesized as previously described in Backes et al.^1^ Figure 1 presents the procedural steps from synthesis to obtaining 3D bioprinted scaffolds. Briefly, 5 g of porcine skin gelatin (#G2500, Sigma-Aldrich) was dissolved in 50 mL of preheated phosphate-buffered saline (PBS) at 50 °C, followed by the dropwise addition of 0.5 mL methacrylic anhydride (MA, #276685, Sigma-Aldrich, USA) under constant stirring. This concentration results in a low methacrylation degree and the formation of low GelMA. GelMA with low MA modification presents low stiffness, making it more cell-friendly to soft tissues such as cartilage and neural tissue.^29,32,33^ The reaction proceeded for 2 h at 50 °C with the temperature monitored every 15 min. The resulting solution was diluted in an equal volume of PBS, transferred into prehydrated dialysis membranes, and dialyzed against distilled water at 40 °C for 5 days with twice-daily water changes. After dialysis, the solution was mixed with 100 mL of ultrapure water, stirred, aliquoted into conical tubes, frozen at −80 °C, and subsequently lyophilized for 2 days. The freeze-dried GelMA was stored at −20 °C until further use.

**Figure 1:**
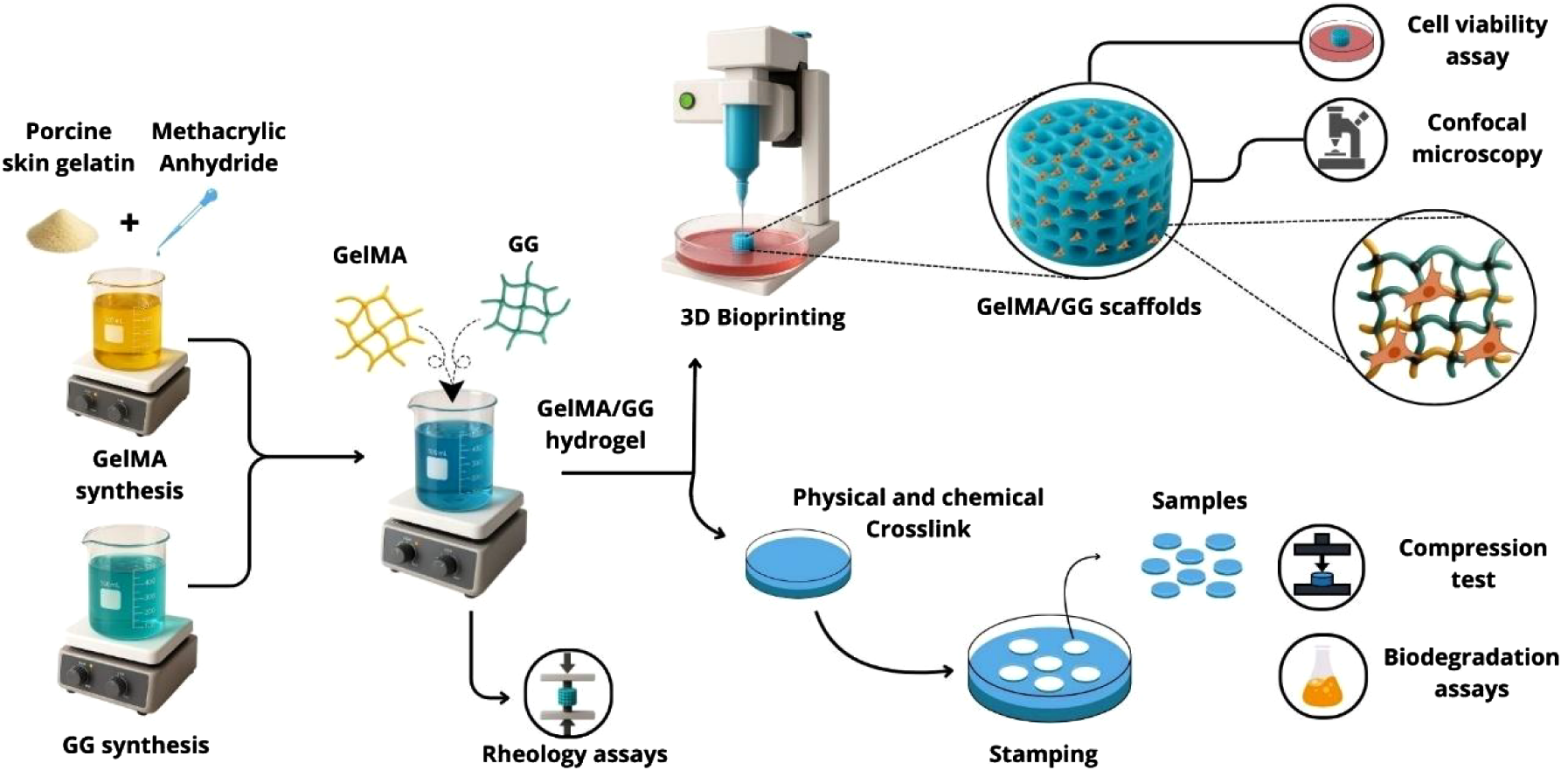
Procedural steps for 3D bioprinting gelatin methacrylate (GelMA)/gellan gum (GG) scaffolds. Designed by the authors with the assistance of Canva’s design platform.

Gellan Gum (GG; Gelzan no. G1910, Sigma-Aldrich) was used without further modification, and the gels were formed by mixing GG with ultrapure water on a magnetic stirrer at 85–90 °C until complete dissolution. At this temperature, the solution became clear and could be stored in a fridge until further use; however, GG has a gel point around 40–65 °C depending on its concentration. In our case, due to low-concentration hydrogels (0.50–0.25 w/v), it could form a clear solution at ca. 40–45 °C.

The hydrogels composed of GG and GelMA were prepared by mixing them in a magnetic stirrer at 40–45 °C, and then used to formulate two concentrations of GelMA (2.5% and 4.0% w/v) and two concentrations of GG (0.25% and 0.50% w/v). The photopolymerization of GelMA was achieved using a photoinitiator (2-hydroxy-4′-(2-hydroxyethoxy)-2-methylpropiophenone, #410896, Sigma-Aldrich) at a concentration of 0.5% in GelMA under UV light exposure for 5 min, followed by a 2 min immersion in PBS.

### 2.2 Characterization

#### 2.2.1 Rheological Characterization

Rheological characterization was performed using a stress-controlled rheometer (Model ARG2, TA Instruments, USA) equipped with a 50 mm parallel plate geometry and a fixed gap of 500 μm. Oscillatory measurements were conducted at 25 °C to determine the viscoelastic properties of the uncross-linked hydrogels. Storage (*G*′) and loss (*G*″) moduli were recorded as a function of angular frequency (ramp-up). The linear viscoelastic region (LVR) was previously determined by using an amplitude sweep test. All tests were conducted with a constant oscillatory strain of 1% and a frequency of 1 Hz.

#### 2.2.2 Mechanical Behavior

The mechanical properties of the hydrogels were determined by compression testing using a universal testing machine (model 6659, Instron, UK) equipped with a 5 N load cell, a 10 mN preload, a deformation rate of 1.3 mm/s, and a testing temperature of 23 °C. The compressive Young’s modulus was calculated using linear regression within the 0.05–0.25% strain range to minimize errors associated with approximating the plate-to-sample contact, and the ultimate compressive modulus was determined at a fixed strain of 20%. Hydrogel samples were fabricated by casting ca. 2.5 mL of hydrogel solution into 35 mm Petri dishes, followed by photopolymerization under UVA light for 5 min (365 nm, 2 mW/cm^2^) and then immersion in PBS for an additional 2 min, as previously described in recent articles published by our group.^4,12^ To minimize attenuation and unwanted optical effects, irradiation was performed with the Petri dish lid removed and at a constant distance of ∼1 cm between the light source and the sample. Cylindrical specimens were obtained immediately after using a 7 mm diameter biopsy punch and gently dried for dimensional measurements: 2.0–2.2 mm in height and approximately 7 mm in diameter.^1^

#### 2.2.3 Degradation Assay

The degradation assay was conducted to assess the material’s stability following both physical and chemical cross-linking. We removed a piece of the untested sample from the sample used for mechanical characterization (∼100 mg). The assay was performed in 24-well culture plates, each containing 1 mL of PBS at room temperature (RT). The PBS was carefully replaced every 2 days by using 1000 μL. To prevent damage to the samples, PBS was added and removed gently from the side of each well. Samples were incubated for predetermined time points: 1, 3, 7, and 14 days at 37 °C under static conditions. At each time point, the samples were carefully retrieved using a spatula, gently dried with paper towels, and weighed using a precision analytical balance with a resolution of 0.001 g. Mass loss (Δ*m* (%)) was calculated using (eq 1), where *m*_i_ is the initial mass and *m*_f_ is the final mass of the sample. Three samples were assessed at each time point.

1 $$\Delta m\left(\%\right)=\frac{\left(m_{i}-m_{f}\right)}{m_{i}}\times 100$$

#### 2.2.4 Morphological Analysis

Hydrogels’ morphological analysis was conducted on freeze-dried samples using a scanning electron microscope (Tescan MIRA FEG, Brno, Czech Republic) operated at 5 kV. The samples were mounted on stubs using double-sided carbon tape, coated with a thin gold layer, and examined to evaluate the surface morphology.

#### 2.2.5 NMR

The methacrylation degree (MD%) of GelMA was quantified by ^1^H-NMR following the procedures reported by Hoch et al.^34^ Approximately 20 mg of either unmodified gelatin or GelMA was dissolved in 0.75 mL of deuterium oxide (D_2_O, 99.9 atom %; Sigma-Aldrich, Steinheim, Germany). Proton NMR spectra were acquired using an NMR instrument (Agilent Technologies 500/54 Premium Shielded, California, USA) operating at 500 MHz. To determine the % MD, the integrated amine proton signals corresponding to methacrylamide-substituted lysine residues were normalized to the aromatic protons of phenylalanine, which served as an internal reference. The degree of substitution was then calculated according to the following relationship (eq 2):

2 $$\mathrm{MD}\;\%=1-\frac{\mathrm{Lysine}\;\mathrm{integration}\;\mathrm{signal}\;\mathrm{of}\;\mathrm{GelMA}}{\mathrm{Lysine}\;\mathrm{integration}\;\mathrm{signal}\;\mathrm{of}\;\mathrm{unmodified}\;\mathrm{gelatin}}\times 100$$

#### 2.2.6 3D Bioprinting and Cell Viability

##### 2.2.6.1 Cell Culture

HUVEC cells were cultured in T75 flasks with Dulbecco’s modified Eagle medium (DMEM) F12 (#12500062, Thermo Fisher, Gibco) supplemented with 10% fetal bovine serum (FBS; #12657011, Gibco) and 1% penicillin/streptomycin (#15070063, Gibco) for 7–10 days until confluence in an incubator at 37 °C and 5% CO_2_. Later, the cells were detached by exposure to 0.25% Trypsin/EDTA at 37 °C for 5 min, followed by enzymatic inactivation with an equal volume of DMEM/F12, and then centrifuged to obtain cell pellets. These cells, with a concentration of 1 × 10^6^ cells, were dispersed in hydrogels to prepare printable bioinks.

##### 2.2.6.2 3D Bioprinting and Cell Viability

Cell viability of HUVECs in GelMA/GG bioink solutions was assessed by using live/dead staining. To this end, the hydrogels of GelMA and GG were filtered using a syringe and a 0.22 μm polypropylene filter (Merck, Millipore) under a laminar flow cabinet at 40 °C. Then, they were gently mixed with a pipet and incubated in a water bath at 37 °C until the HUVECs were resuspended in the hydrogels using an up-and-down movement.

After centrifugation, the supernatant was removed, and the cells were carefully resuspended in the as-prepared GelMA/GG hydrogel (at a concentration of 10^6^ cells/mL of bioink) to prepare the bioprintable bioinks. Using a pipet, the bioink was then transferred to a 5 mL plastic syringe. Air bubbles were carefully removed, and a 22-G nonbeveled needle (BD, Brazil) was attached. 3D bioprinting of the bioinks was performed in a 35 mL Petri dish, followed by the same cross-linking protocol (UV and PBS). The bioprinter (model Octopus, 3D Biotechnology Solutions, Brazil) was operated in a flow chamber at RT (20–22 °C) under the following conditions: nozzle temperature of 37 °C, heating bed off, and speed of 5 mm/s. We produced a circular construct with dimensions of 8 mm (diameter) × 2 mm (height) and a layer height of 0.2 mm (cross-hatched). Afterward, the samples were photopolymerized under UVA light (365 nm, 2 mW/cm^2^) for 5 min and transferred gently with a spatula to a 48-well plate. DMEM F12 was then brought up to 1 mL, and the samples were incubated at 37 °C with 5% CO_2_. The cell media was changed every 2–3 days. Additional details on cell handling during bioprinting preparation, G-code generation, and bioprinter operation are provided in a protocol recently published by our group.^1^

After 1, 7, and 14 days, the bioprinted samples were retrieved and transferred to confocal dishes using a spatula, washed with PBS at room temperature, and incubated with a 1:10 (v/v) dilution of live/dead reagent (no. R37601, Thermo Scientific) in culture medium for 10 min at 37 °C. To preserve cell viability and imaging quality, samples were protected from light and sealed with parafilm to prevent drying. Imaging was conducted using confocal microscopy (Leica TCS SP8) with 488 nm (staining live cells, calcein AM) and 594 nm (staining dead cells, propidium iodide) lasers. Cell viability was quantified by separating the green and red channels and calculating the occupied areas in each channel using ImageJ (NIH, USA). Three images (1024 × 1024 pixels) from 3 different samples were analyzed for each bioink tested.

#### 2.2.7 Statistical Analysis

Data are presented as mean ± standard deviation and analyzed using the one-way analysis of variance (ANOVA), nonparametric test (without Gaussian distribution of residuals), followed Dunn’s multiple comparisons post hoc test. Statistical significance was set at *p* < 0.05 and is denoted in the figure’s caption.

## 3 Results and Discussion

### 3.1 Rheological and Mechanical Characterizations

The bioinks used in the fabrication of bioprinted structures serve multiple functions, including preserving cellular phenotype, directing cell fate, and recapitulating tissue characteristics, such as anisotropy or spatially varying properties from soft tissues.^35^ To achieve these features, the resulting scaffolds must exhibit key properties, including biocompatibility, controlled biodegradability, suitable rheological behavior for printability, and mechanical characteristics that mimic those of the target tissue.^36^

GelMA offers the flexibility to adjust its mechanical and rheological properties by controlling the methacrylate and cross-linking degrees, making it an appropriate choice for bioprinting applications.^3,27^ Still, achieving good printing with adequate mechanical properties is challenging due to poor GelMA printability and its rapid biodegradation at low concentrations and low methacrylate degrees.^3^ Therefore, mixing GelMA with another biocompatible hydrogel that hinders fast biodegradation and also offers higher printability enables the biofabrication of tailored hydrogels with enhanced printing fidelity, high cell survival, and adequate mechanical properties.^19^

Critical rheological parameters such as shear-thinning behavior, viscosity, and viscoelastic properties govern the material’s ability to flow through the printer nozzle and retain its structure after deposition.^37^ An ideal bioink should display low viscosity under shear to facilitate smooth extrusion, while rapidly regaining its structure postprinting to preserve printed geometry.^37^ Moreover, suitable viscoelastic properties are vital for supporting encapsulated cells, promoting uniform distribution, and preventing excessive mechanical stress that could lead to cell death.^38^ Therefore, rheological evaluation is essential for tuning bioink formulations to ensure cell survival coupled with printing conditions and biological requirements, ultimately enhancing the reproducibility and functional performance of bioprinted tissue constructs.^22^

Figure 2a shows complex viscosity (η*) versus angular frequency, indicating that all hydrogel formulations exhibit pronounced pseudoplastic shear-thinning behavior, with η* decreasing with increasing angular frequency. This rheological behavior is highly desirable for extrusion-based bioprinting as it facilitates smooth flow through the nozzle under lower shear stress and promotes rapid structural recovery after deposition. The combination of GelMA and GG demonstrated a clear dependence of viscosity on the polymer concentration. Especially, samples containing 0.50 wt %. GG showed higher viscosity across the analyzed frequencies than did 0.25 wt % GG. This trend aligns with previous findings that attribute the increased viscosity to GG’s ability to form a strong physically cross-linked network, thereby contributing to a more entangled and robust hydrogel structure under low shear conditions.^39^

**Figure 2:**
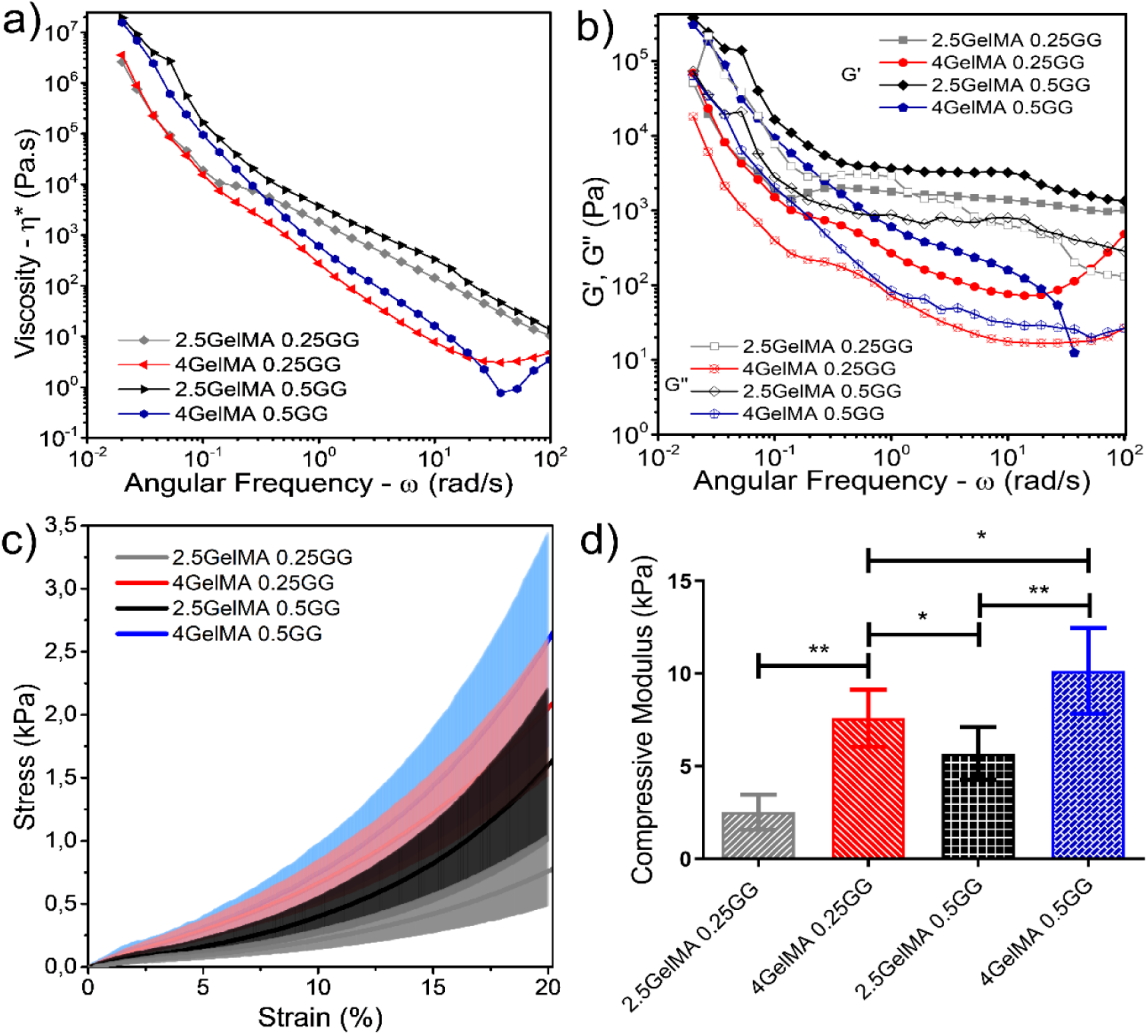
Mechanical properties of gelatin methacrylate (GelMA)/gellan gum (GG) hydrogel bioinks. a) Frequency sweep analysis of GelMA/GG hydrogels showing complex viscosity (η*) as a function of angular frequency (ramp-up) (ω); b) *G*′ and *G*″ versus angular frequency (ramp- up) for GelMA/GG hydrogels; c) stress versus strain curves during compression testing; and d) Young’s modulus for GelMA/GG hydrogels (*n* = 6, **p < 0.005, *p < 0.05).

Moreover, increasing the GelMA concentration from 2.5% to 4.0% resulted in two distinct behaviors, depending on the GG content. In systems with low GG concentration, the higher GelMA content slightly increased η*, as it contributed to the chemical cross-linking density and mechanical resilience of the hydrogel.^40^ On the other hand, at 0.25 wt % GG concentrations, GG played a more dominant role in the early gelation, while the higher GelMA acted more as a lubricant in the system. In this case, η* presented only minor reductions at low angular frequencies but with a steeper slope, leading to lower η* values at higher angular frequencies compared to all other systems.

This behavior suggests the formation of a dual network system in which the balance between the GG physical network and the GelMA chemical interactions governs the viscoelastic response. Under high-shear conditions, this interplay may also favor partial decoupling of the phases, contributing to the observed reduction in viscosity. According to Mouser et al.,^29^ incorporating GG enhances yield stress and filament formation, which are critical for maintaining structural fidelity during and after bioprinting. These findings are further supported by Martorana et al.,^30^ who observed that GG-based bioinks display pseudoplastic behavior and improved extrusion properties, particularly when combined with functional additives or cross-linkers. Therefore, the rheological data in Figure 2 align well with the existing literature, reinforcing the role of both GG and GelMA in tailoring viscosity profiles to facilitate the fabrication of bioprintable constructs.

Figure 2b presents *G*′ and *G*″ through frequency sweep curves for the GelMA/GG formulations tested. All of them showed *G*′ exceeding *G*″ throughout the entire frequency range, confirming the predominance of solid-like behavior even without chemical/physical cross-linking, an essential feature for maintaining construct fidelity after deposition in 3D bioprinting.

Recent studies have shown that the ability of hydrogels to retain their shape after extrusion is also strongly influenced by their viscoelastic properties, specifically *G*′ and *G*″. These moduli quantify the material’s elastic and viscous responses, respectively, and are recognized as key indicators of shape fidelity and construct stability in bioprinting. For instance, a high storage modulus has been correlated with improved postextrusion shape retention, while the loss modulus provides information on dissipation and flows under shear.^41^

As expected, both *G*′ and *G*″ increased with higher GG concentrations, indicating a stiffer, more cohesive network; however, increased GelMA content exerted a lubricating effect, significantly decreasing the level of *G*′. This is consistent with previous findings by Koivisto et al.,^42^ who demonstrated that GG-rich hydrogels enhanced mechanical integrity due to physical cross-linking and chain entanglements, which reinforce the hydrogel’s elasticity.

In the system with a low GG concentration (0.25% v/w), the addition of higher GelMA contents led to a less elastic response, as evidenced by the reduction in *G*′, although the curve exhibited a final increase, consistent with GelMA being a lower-viscosity component. Interestingly, *G*″ in these samples showed an unexpected trend, shifting from the highest to the lowest value upon the addition of 2.5% GelMA. Conversely, in the system with 0.50% GG, the formulation containing 2.5% GelMA displayed the highest *G*′, while further GelMA addition altered this behavior, likely due to its lubrication effect, as supported by the reduction in *G*″. Overall, increasing the GelMA concentration from 2.5 to 4.0% elevated both moduli, particularly at higher frequencies, with more pronounced effects in systems containing low GG concentrations.

In summary, rheological characterization revealed that both the viscosity and viscoelastic moduli of the GelMA/GG hydrogels were strongly dependent on the hydrogel concentration. Higher GG and GelMA contents enhanced shear-thinning behavior, increased complex viscosity, and reinforced the elastic and viscous moduli, supporting the formation of a dual physical–chemical network. Altogether, these findings reinforce that fine-tuning the GG and GelMA ratios enables precise modulation of viscoelastic properties, allowing for adequate printability and structural stability without compromising the material’s ability to support cell viability.

Figures 2c,d shows the stress versus strain curves and the compressive elastic modulus of a series of developed GelMA/GG hydrogels. Both systems underwent UV-induced photo-cross-linking, combined with chemical and thermally driven gelation. Photo-cross-linking cross-linked GelMA, while PBS, which contains Ca^2+^ ions, can cross-link GG components. Coutinho et al.^27^ performed an experiment using methacrylated GG (MeGG) hydrogels, alternating their exposure between water and PBS solutions. The authors noted that the hydrogels expanded in water, contracted when placed in PBS, and then expanded again upon returning to water. This experiment validated the ionic characteristics of the swelling–deswelling behavior of the MeGG hydrogels. It also demonstrated the ability to manipulate the physical characteristics of the produced MeGG hydrogels by altering the solution in which they were submerged. A similar trend was also observed in alginate hydrogels.^43^

The GelMA/GG systems consisted of two GG concentrations (0.25 and 0.50% w/v) combined with either 2.5 or 4.0% (w/v) GelMA. At fixed GelMA content, increasing GG concentration significantly raised the compressive modulus, from 2.5 ± 0.9 kPa to 5.7 ± 1.4 kPa (*p* < 0.005), as can be seen in the upward-shifting trend in Figure 2c. Similarly, for the 2.5% (w/v) GelMA formulation, the compressive modulus increased from 7.5 ± 1.5 kPa to 10.1 ± 2.3 kPa for the 4.0% (w/v) GelMA formulation. Similarly, by maintaining the GG concentration at 0.25% (w/v) or 0.50% (w/v), as the GelMA concentration was increased from 2.5 to 4.0% (w/v), similar increases were observed in the Young’s moduli.

The hydrogels were tested to 20% strain without failure, and results showed that, at a fixed GelMA content of 2.5% (w/v), increasing the GG concentration significantly increased the ultimate compressive strength from 0.7 ± 0.3 kPa to 1.6 ± 0.6 kPa. For formulations containing 4.0% (w/v) GelMA, increasing the GG content improved compressive strength from 2.0 ± 0.5 kPa to 2.6 ± 0.8 kPa. These results demonstrated that tuning the concentrations of GelMA and GG enabled the obtention of distinct mechanical properties capable of matching the characteristics of the target tissue.

The rheological and mechanical behavior of uncross-linked (rheological) and cross-linked (mechanical) systems evidenced the complex interactions of dual network system, where the GelMA component mainly exists as physically entangled gelatin chains within the GG network. By increasing the GelMA concentration, since the MD is low (∼20%), more uncross-linked gelatin chains were introduced, which acted as plasticizers, thus reducing the overall physical network density and chain entanglement, thereby leading to a softer ink with a lower storage modulus (*G*′; Figure 2a,b). Therefore, systems with a higher GelMA but low GG content exhibited lower complex viscosities at higher angular frequencies, reflecting reduced physical interactions, and enhanced chain mobility. However, after photo-cross-linking, the situation reverses. The methacrylate groups on GelMA underwent covalent cross-linking, forming a dense chemical network. Higher GelMA content thus translated to a higher density of covalent cross-links, resulting in a stiffer hydrogel with significantly increased compressive modulus.

Furthermore, longer UV exposure times can improve GelMA cross-linking and reduce its dissolution; however, this approach is limited by the methacrylation degree (%MD). The fabricated GelMA in this study presented an MD of ∼20%, consistent with low GelMA^44^ (Supplementary Figure S1). Likewise, using stronger cross-linking agents, such as CaCl_2_, and longer immersion times can further cross-link GG. However, high concentrations of CaCl_2_ disrupt various intracellular mechanisms, triggering apoptosis.

Sahranavard et al.^8^ investigated the gelling properties of GG at different CaCl_2_ concentrations and combined dual cross-linking with glutaraldehyde, assessing their potential for bioprinting. The hydrogel of GG (3% w/v), containing 0.5% (w/v) CaCl_2_ and 2% (v/v) glutaraldehyde, showed excellent printing fidelity and around 80% cell viability after 5 days of cell culture and a superior Young’s modulus of 19 ± 2 kPa. Similarly, a hydrogel cross-linked only with CaCl_2_ showed a Young’s modulus of 8 ± 3 kPa, demonstrating the role of cross-linking mechanisms in mechanical stability and printability.^8^

### 3.2 Biodegradation Assay

Biodegradation assays were performed to assess the stability of the hydrogels under simulated conditions, specifically by immersion in PBS and incubation at 37 °C (Figure 3a). 0.2 GG2.5GelMA samples underwent significant weight loss even on the first day of analysis, exhibiting a weight loss of 29 ± 7%, while 4GelMA0.25GG, 2.5GelMA0.5GG, and 0.5GG4GelMA showed 13 ± 4%, 17 ± 6%, and 16 ± 3%, respectively. The increase in the concentration of GelMA for the composition 4GelMA0.25GG resulted in a reduction of less than half of the initial weight loss due to a higher dual network system between the two systems. Conversely, it could be observed that in hydrogels with 0.50% (w/v) GG, the GelMA content did not change significantly, as these systems already have a more stable network. Over 3, 7, and 14 days, all the compositions proceeded with increasing weight loss due to the dissolution of uncross-linked components; e.g., the composition 4GelMA0.5GG, which initially presented a stable behavior, showed higher weight loss at the end of 14 days (28 ± 2%, compared to 21 ± 8% of 2.5GelMA0.5GG) due to a higher GelMA content. The GelMA utilized in this study demonstrated a low methacrylation level (approximately 20%). Consequently, not all gelatin binding sites were modified with methacrylate groups and could not cross-link under photo-cross-linking conditions after a 5 min exposure, as illustrated in Figure S1. Furthermore, a significant fraction of gelatin could undergo physical dissolution upon exposure to PBS, and this degradation was more pronounced in systems with higher GelMA concentrations. Samples of 2.5GelMA0.25GG and 4GelMA0.25GG at 14 days exhibited weight losses of 42 ± 4% and 24 ± 4%, respectively, highlighting the influence of GG as a network-anchoring agent, capable of forming stronger networks and hindering biodegradation. Figure 3b displays SEM micrographs of freeze-dried cross-linked hydrogels, revealing two distinct groups based on GG content. At lower concentrations of GG and GelMA, the hydrogel foam shows larger pores. In contrast, at higher additions of either GelMA or GG, the hydrogel yields a smaller, more uniform set of pores.

**Figure 3:**
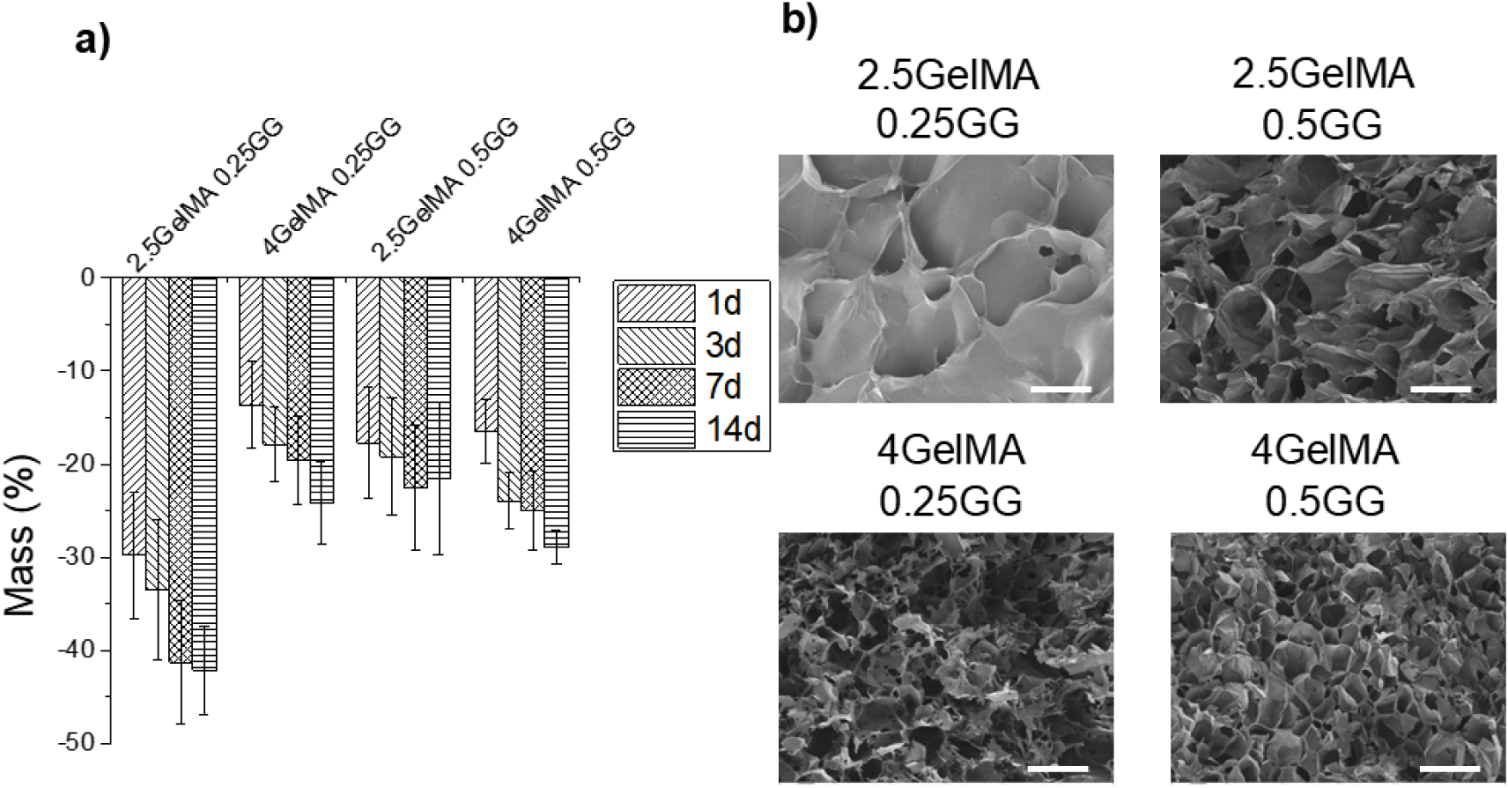
(a) Mass variation versus time for gellan gum (GG)/gelatin methacrylate (GelMA) cross-linked printed hydrogels, presenting varied relative compositions. b) SEM micrographs of freeze-dried hydrogels (scale 500 μm).

### 3.3 Bioprinting and Cell Viability

Figure 4a illustrates the 3D bioprinting process, where the moving needle is depositing the first layer of the hydrogels, as shown in Video S1.

**Figure 4:**
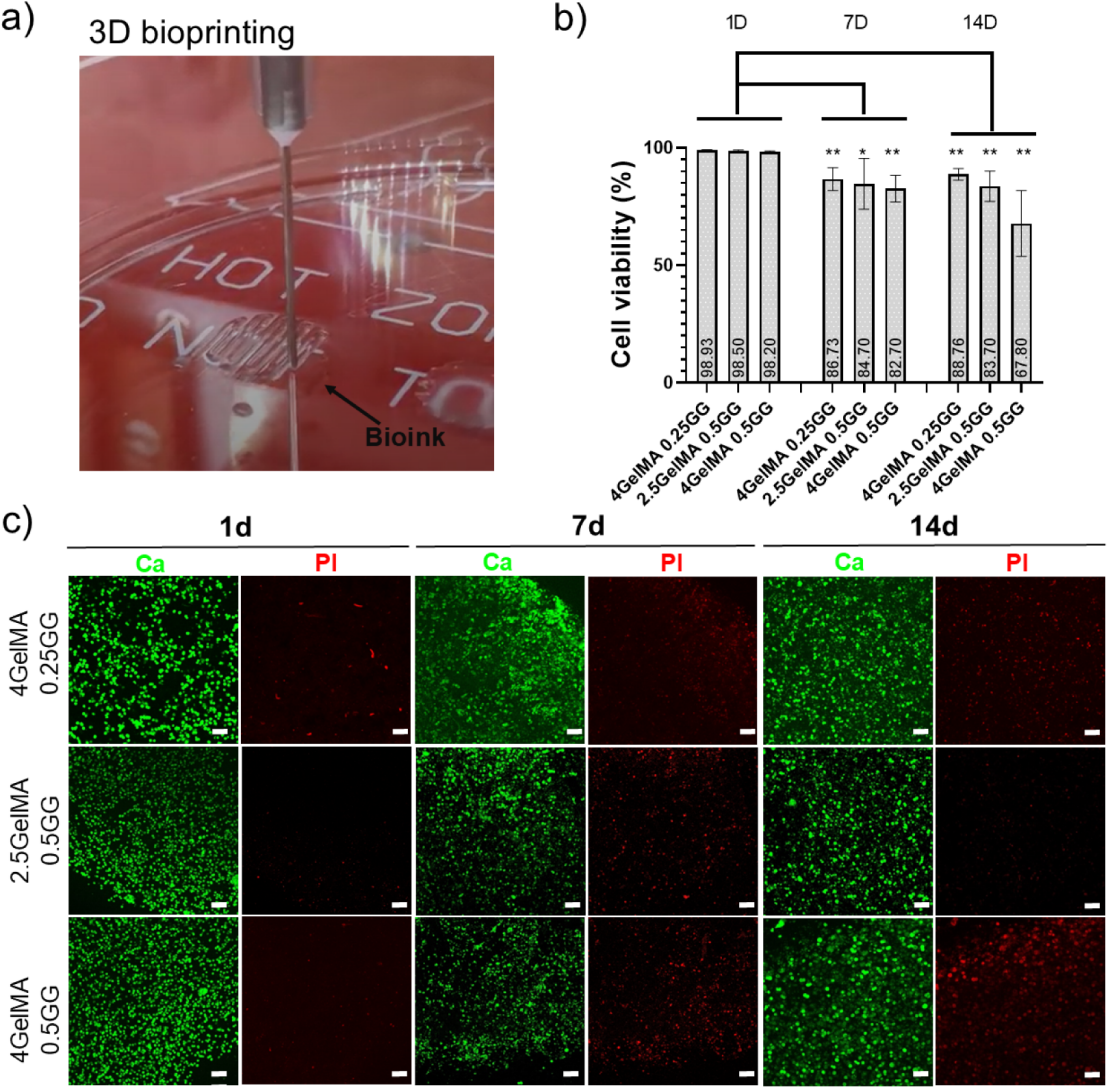
Bioprinted gelatin methacrylate (GelMA)/gellan gum (GG) bioinks: a) 3D bioprinting process depositing the first layer of the hydrogels (see Supplementary Video 1) (author’s personal collection); b) quantification of cell viability (live/dead) for the various compositions during different culture times; and c) confocal micrographs of the live/dead assay for the bioprinted hydrogels under different culture times (scale bars: 100 μm), **p < 0.005, *p < 0.05).

The HUVEC cell line was employed in this study as a well-established and robust model cell line to evaluate the cytocompatibility of the bioink and to validate the bioprinting process, including cell survival and morphology following printing. Its use was not intended to demonstrate lineage-specific differentiation but rather to provide a reliable biological benchmark for assessing ink–cell interactions, print fidelity, and postprinting viability. Given their reproducible growth characteristics and sensitivity to microenvironmental changes, HUVECs are widely accepted in biomaterials research as a suitable in vitro model for preliminary biocompatibility and process validation studies.

Figure 4b shows the corresponding cell viability data (live/dead) assay, and Figure 4c presents the confocal micrographs of the GelMA/GG bioprinted hydrogels. Due to high biodegradation, low mechanical properties, and difficulties in bioprinting, we decided not to proceed with characterizing the composition 2.5GelMA/0.25GG.

The cell viability analysis revealed a time-dependent decrease across all formulations. For 4GelMA0.25GG, values at day 1 were significantly higher than those at days 7 and 14 (*p* < 0.01), indicating a marked decline followed by stabilization thereafter (NS comparison between days 7 and 14). Similarly, 2.5GelMA0.5GG showed a more pronounced reduction from day 1 to day 7 (*p* < 0.05) and day 14 (*p* < 0.01), where it plateaued over the 7–14 day period (NS comparison between days 7 and 14). The most pronounced reduction occurred in 0.5GG 4GelMA, where all comparisons involving day 1 were highly significant, indicating a continuous and substantial decrease throughout 7 and 14 days (*p* < 0.01). Collectively, these results demonstrate that all hydrogels underwent significant degradation or reduction in cell viability over time, with the 4GelMA0.5GG formulation exhibiting the steepest temporal decline. In contrast, the 4GelMA0.25GG formulation maintained relatively higher stability after the first week.

A plausible explanation for this phenomenon, observed over longer culture periods such as 7 and 14 days, may be the formation of a denser, stronger, and more robust dual network system within the hydrogel framework encapsulating the cells, thereby inhibiting nutrient transport and proliferation. As GelMA underwent photo-cross-linking and gelatin contributed to ionic or physical gelation, the resulting network became increasingly restrictive. This increased restriction could limit the diffusion of vital nutrients and oxygen to the encapsulated cells while concurrently impeding the removal of metabolic waste and limiting cell spreading.^45^

This diffusional barrier is especially critical for cells situated in the interior regions of the construct, where mass transport limitations are most pronounced. Additionally, higher polymer concentrations enhance hydrogel stiffness, which, although advantageous for mechanical stability and structural integrity in bioprinting, may impose mechanical stress on encapsulated cells, potentially affecting cell migration, cytoskeletal organization, mechanotransduction pathways, and cell fate.^46^

The present study successfully demonstrated the feasibility, cytocompatibility, and printability of the GelMA/GG bioinks; however, several complementary characterizations remain to be explored. Although the biodegradation behavior was assessed, future work will include a systematic evaluation of the swelling ratio to elucidate network density and fluid uptake dynamics. In addition, in situ rheological studies of gelation kinetics during UV irradiation are planned to provide quantitative insight into cross-linking rates and guide formulation optimization. While the current bioink exhibited qualitative printability and shape fidelity (Supporting Information, Video 1), forthcoming studies will implement quantitative printability assessments, including line width spreading and filament homogeneity. Moreover, the biodegradation in PBS provides a foundational baseline; yet, for translational relevance, degradation under enzymatic conditions (e.g., collagenase and lysozyme) will be investigated. Finally, although high postprinting cell viability was achieved, subsequent analyses will incorporate immunofluorescence staining (e.g., phalloidin/DAPI) to characterize cell morphology, spreading, and cytoskeletal organization within the cross-linked matrix.

## 4 Conclusions

The gelatin methacrylate (GelMA)/gellan gum (GG)-based bioinks developed in this study effectively combine the complementary properties of their components, enabling the design of functional scaffolds with tunable mechanical properties, predictable biodegradation behavior, and cell biocompatibility—key requirements for successful tissue engineering applications. The incorporation of GG into the hydrogel formulations enhanced the elastic component, as shown by rheological characterization. Additionally, GG contributed to mechanical reinforcement and delayed biodegradation by forming a physically cross-linked network that complemented GelMA’s photo-cross-linkable matrix. This synergistic interaction yielded a more robust dual-network system capable of mimicking the mechanical environment of soft tissues, such as cartilage and the central nervous system, with Young’s moduli within the desired range (0.1–10.0 kPa). These bioinks presented adequate rheological behavior to be bioprinted, resulting in high cell viability at 1 day (>98%) and maintaining high viability (>85%) for 4GelMA0.25GG and 2.5GelMA0.5GG during longer culture times, such as 14 days. These findings support the potential of GelMA/GG hydrogels as versatile, tunable bioinks for cartilage, and soft tissue engineering.

## Supplementary Material





## Data Availability

Data used are available throughout the manuscript text.

## Notes

Figure S1: NMR spectra for gelatin and gelatin methacrylate (GelMA) (PDF)
3D bioprinting process occurring where the moving needle is depositing the first layer of the hydrogels (MP4)
