# Supplementary material for: Balancing Strength and Cell Viability in Gelatin Methacrylate/Gellan Gum Bioink Formulations
Source: ACS Omega. 2026 Mar 3;11(10):15911–21. doi: 10.1021/acsomega.5c09665 (PMC13000567; doi:10.1021/acsomega.5c09665)
Supplement: Supplementary file 1 [file ao5c09665_si_001.pdf]

Balancing Strength and Cell Viability in Gelatin Methacrylate/Gellan Gum bioinks Formulations

Eduardo H Backes[1-2*], Leonardo A Pinto[1], João F G Neto[1], Tainara P Lima Lima[1], Pedro L Granja[3]; Luiz A Pessan[1-2], Marimélia A Porcionatto[4-5]

1. Federal University of São Carlos (UFSCar), Graduate Program in Materials Science and Engineering (PPGCEM),13565-905, São Carlos, Brazil
2. Federal University of São Carlos (UFSCar), Department of Materials Engineering, 13565-905, São Carlos, Brazil
3. Instituto de Investigação e Inovação em Saúde (i3S), i3S - Universidade do Porto/Biofabrication Group, 4200-135, Porto, Portugal.
4. Universidade Federal de São Paulo (UNIFESP), Laboratory of Molecular Neurobiology, Escola Paulista de Medicina, 04023-062, São Paulo, Brazil
5. Universidade Federal de São Paulo (UNIFESP), Department of Biochemistry, Escola Paulista de Medicina, UNIFESP, 04023-062, São Paulo, Brazil

*corresponding author: backes@ufscar.br

The GelMA developed in this research exhibited a methacrylation degree (MD) of approximately 20%, consistent with the low GelMA formulations reported in the literature[1]. The MD was assessed using proton nuclear magnetic resonance (^1H NMR) spectroscopy. In summary, we calculated the percentage of methacrylation by the integrated signals of amine protons associated with methacrylamide-substituted lysine residues (usually observed at ~5.3–5.7 ppm for the vinyl protons), which were normalized against the aromatic proton signals of phenylalanine residues (seen at ~7.2–7.4 ppm).

Formulations of low-MD GelMA result in lower crosslinking density during photopolymerization and typically produce softer hydrogels, higher swelling ratios, and greater cell viability compared to medium- or high-MD GelMA alternatives. This degree of modification is especially appropriate for applications that demand improved cell spreading, movement, and matrix restructuring while ensuring adequate structural stability for three-dimensional bioprinting and tissue engineering.

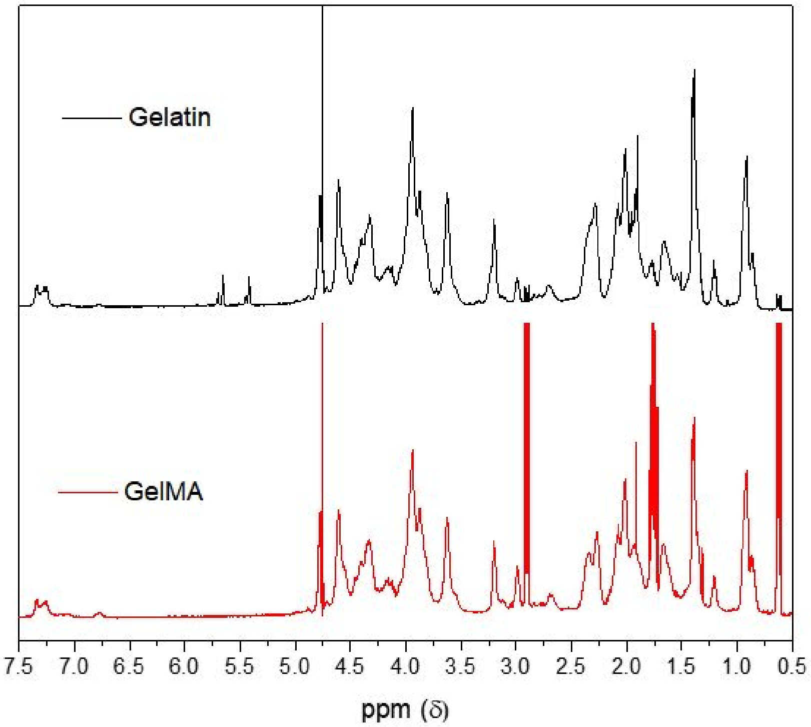

Figure S1: NMR spectra for Gelatin and gelatin methacrylate (GelMA).

Reference

(1) Nichol, J. W.; Koshy, S. T.; Bae, H.; Hwang, C. M.; Yamanlar, S.; Khademhosseini, A. Cell-Laden Microengineered Gelatin Methacrylate Hydrogels. *Biomaterials* 2010, *31* (21), 5536–5544
